# Causal Inference in the Presence of Interference in Sponsored Search Advertising

**DOI:** 10.3389/fdata.2022.888592

**Published:** 2022-06-21

**Authors:** Razieh Nabi, Joel Pfeiffer, Denis Charles, Emre Kıcıman

**Affiliations:** ^1^Department of Biostatistics and Bioinformatics, Emory University, Atlanta, GA, United States; ^2^Microsoft Research, Redmond, WA, United States; ^3^Microsoft Corporation, Redmond, WA, United States

**Keywords:** causal inference, allocational interference, spillover effect, dependent data, counterfactual layout, online advertising

## Abstract

In classical causal inference, inferring cause-effect relations from data relies on the assumption that units are independent and identically distributed. This assumption is violated in settings where units are related through a network of dependencies. An example of such a setting is ad placement in sponsored search advertising, where the likelihood of a user clicking on a particular ad is potentially influenced by where it is placed and where other ads are placed on the search result page. In such scenarios, confounding arises due to not only the individual ad-level covariates but also the placements and covariates of other ads in the system. In this paper, we leverage the language of causal inference in the presence of interference to model interactions among the ads. Quantification of such interactions allows us to better understand the click behavior of users, which in turn impacts the revenue of the host search engine and enhances user satisfaction. We illustrate the utility of our formalization through experiments carried out on the ad placement system of the Bing search engine.

## 1. Introduction

In recent years, advertisers have increasingly shifted their ad expenditures online. One of the most effective platforms for online advertising is search engine result pages. Given a user query, the search engine allocates a few ad slots (e.g., above or below its organic search results) and runs an auction among advertisers who are bidding and competing for these slots. Quantifying the effectiveness of ad placement is vital not only to the experience of the user, but also revenue of the advertiser and the search engine. Click yield is a common metric used in this regard. Often, statistical and flexible machine learning models are used to predict the click behavior of users by estimating the likelihood of receiving a click in a given slot using logged data. A rich literature is devoted to click prediction in sponsored search advertising (Shaparenko et al., 2009; Cheng and Cantú-Paz, 2010; Cheng et al., 2012; Xiong et al., 2012; Zhang et al., 2014; Nabi-Abdolyousefi, 2015; Effendi and Ali, 2017; Bisht and Susan, 2021). For a survey on click prediction in online advertising please refer to Wang (2020). However, a comprehensive understanding of click behavior requires causal, rather than associative, reasoning (Bottou et al., 2013; Yin et al., 2014; Hill et al., 2015; Zeng et al., 2021).

Causal inference is central to making data-driven decisions. Inferring valid cause-effect relations, even with granular data and large sample sizes, is complicated by confounding induced by common causes of observed exposures and outcomes. In classical causal inference, it is assumed that samples are *independent and identically distributed* (iid). However, a causal view of ad placement under the iid assumption is implausible as ads interfere with one another from the beginning of the auction until the end when clicks on impressed ads are recorded. In non-iid settings, confounding arises due to not only the individual ad-level covariates but also the exposures and covariates of other ads in the system. This is commonly referred to as *interference* (Hudgens and Halloran, 2008). Incorporating knowledge of interference into the statistical models used to compute rank scores for each ad can help optimize the final layout of each search page. Moreover, a proper understanding of the interference issue in relation to causal inference directly impacts engineering of more purposeful interventions and design of more effective A/B testing for ad placement. Alternatively, randomized experiments via bipartite graphs offer a useful formalism to study two-sided market experiments under violation of iid assumption (Pouget-Abadie et al., 2018, 2019; Bajari et al., 2021; Harshaw et al., 2021; Johari et al., 2022). This stands in contrast with interference that occurs on networks where all units are of the same type (e.g., ads in a block)—in bipartite experiments, there is a distinction between units that can be subject to an intervention and units whose responses are of interest to the experimenter. Hence, modeling what we are after in the context of sponsored search advertising is closer to the causal framework for modeling interference in social networks.

In this paper, we formalize the problem of interference among ads using the language of causal inference. To the best of our knowledge, this is the first analyis of ads under the plausible and realistic setting of interference. We hope our proposed framework serves as a benchmark for future work in search advertising that go beyond the classical iid assumption. Throughout the paper, we discuss mechanisms that give rise to interference in ad placement. Using graphical models, we assume a causal structure that encodes the various sources of interference. We formulate our causal questions and discuss the identification and estimation of relevant effects. Our experiments find statistically significant interference effects among ads. We further adapt the *constraint-based* structure learning algorithm *Fast Causal Inference* (Spirtes et al., 2000) to verify the correctness of our presumed causal structure and learn the underlying mechanisms that give rise to interference. Finally, we incorporate the knowledge of interference to improve the performance of the statistical models used during the course of the auction. We demonstrate this improvement in performance by running experiments that closely resemble the framework in the Genie model—an offline counterfactual policy estimation framework for optimizing Sponsored Search Marketplace in Bing ads (Bayir et al., 2019).

## 2. Preliminaries and Setup

In causal inference, we are interested in quantifying the cause-effect relationships between a treatment variable *A* and an outcome *Y* using experimental or observational data. A common setting assumes that the treatment received by one unit does not affect the outcomes of other units—this is known as the *stable unit treatment value assumption* or SUTVA Rubin (1980) and is informally referred to as the “no-interference” assumption. In this setting, the *average causal effect (ACE)* of a binary treatment *A* on *Y* is defined as *ACE*: = 𝔼[*Y*(1)] − 𝔼[*Y*(0)], where *Y*(*a*) denotes the counterfactual/potential outcome *Y* had treatment *A* been assigned to *a*, possibly contrary to the fact.

Causal inference uses assumptions in causal models to link the observed data distribution to the distribution over counterfactual random variables. A simple example of a causal model is the *conditionally ignorable model* which encodes three main assumptions: (i) *Consistency* assumes the mechanism that determines the value of the outcome does not distinguish the method by which the treatment was assigned, as long as the treatment value assigned was invariant, (ii) *Conditional ignorability* assumes *Y*(*a*) ⊥ *A* ∣ *X*, where *X* acts as a set of observed confounders, such that adjusting for their influence suffices to remove all non-causal dependence between *A* and *Y*, and (iii) *Positivity* of *p*(*A* = *a* ∣ *X* = *x*), ∀ *a, x*. Under these assumptions, *p*[*Y*(*a*)] is identified as the following function of the observed data: $\underset{X}{\sum }p\left(Y|A=a,X\right)\times p\left(X\right),$ known as *backdoor adjustment* or *g-formula* (Robins, 1986; Pearl, 2009). For a general identification theory of causal effects in the presence of unmeasured confounders see (Huang and Valtorta, 2006; Shpitser and Pearl, 2006; Bhattacharya et al., 2020a). Alternative causal quantities of interest include conditional causal effects (effects within subpopulations defined by covariates) (Shpitser and Pearl, 2012), mediation quantities (which decompose effects into components along different mechanisms) (Shpitser, 2013), and the effects of decision rules in sequential settings (such as dynamic treatment regimes in personalized medicine) (Nabi et al., 2018, 2019).

In this paper, we relax the implausible assumption of no-interference in ad placement. Interference among ads across different pageviews creates the most extreme scenario of *full interference*, as this allows for user interaction with the system over multiple time frames. Following the convention in Sobel (2006), Hudgens and Halloran (2008), Tchetgen and VanderWeele (2012), and Ogburn and VanderWeele (2014), we model only interference within pageviews and restrict any cross-pageview interference among ads. In other words, we restrict the interference to spatial constraints and exclude temporal dependence across pageviews. This is known as *partial interference* and could be justified by the fact that pageviews are query specific and are separated by time and space. In presence of interference, the counterfactual *Y*(*a*) is no longer well-defined as we need to distinguish ads by a proper indexing scheme and consider the treatment assignments of other ads simultaneously.

Suppose we have *N* pageviews, indexed by *n* = 1, …, *N*, with each containing *m* impressed ads. We index the ads on each pageview by *i* = 1, …, *m* based on the order in which they appear on the page. The *i*-th ad on the *n*-th pageview is represented by the tuple (*X*_*ni*_, *A*_*ni*_, *Y*_*ni*_), where *X*_*ni*_ denotes the vector that collects all the ad-specific features such as geometric features (e.g., line width, pixel height), decorative features (e.g., rating information, twitter followers), and other textual features extracted from the ad. *A*_*ni*_ denotes the treatment and is predefined by the analyst. An example of a treatment is the *block* membership of the ad: an indicator that specifies whether the ad is placed on top of the page (Top) or bottom of the page (Bottom). Ads can also appear elsewhere such as the sidebars. In this paper, without loss of generality, we assume we only have two distinct blocks of ads on each pageview: Top and Bottom. *Y*_*ni*_ denotes a binary indicator of receiving a click by the user. We denote the state space of a random variable *V* by 𝔛_*V*_.

Let **X**_*n*_: = (*X*_*n*1_, …, *X*_*nm*_), **A**_*n*_: = (*A*_*n*1_, …, *A*_*nm*_), and **Y**_*n*_: = (*Y*_*n*1_, …, *Y*_*nm*_) collect the features, treatment assignments, and outcomes of all the ads on the *n*-th pageview, respectively. We define the counterfactual *Y*_*ni*_(**a**_*n*_) to be the click response of the *i*-th ad on the *n*-th pageview where every ad on the same pageview is relocated according to the treatment assignment rule **a**_*n*_, which is a vector of size *m* and the *i*-th element *a*_*i*_ denotes the treatment value of the *i*-th ad. This notation makes the interference among ads on the same pageview more explicit as the potential outcome of a single ad now depends on the entire treatment assignment **a**_*n*_, rather than just *a*_*ni*_. The causal effect of interventions in the presence of interference can be quantified by comparing such counterfactuals under different interventions; for instance *Y*_*ni*_(**a**_*n*_) vs. $Y_{ni}(a\prime_{n}),$ where **a**_*n*_ and $a\prime_{n}$ denote two plausible interventions.

In the next section, we discuss various sources that give rise to interference among ads and propose a causal graphical model that captures such interactions in a reasonable way. In what follows, we discuss various ways of quantifying the interference effect among ads and provide sufficient conditions for identification of such effects along with estimation strategies. In general, we observe fewer pageviews that would have *m* > 5 impressed ads. This may affect the finite sample performances of our effect estimations for such pageviews, discussed in Section 4.3. The number of impressed ads does not affect any of our identification claims in Section 4.2. We do consider pageviews with up to *m* = 8 impressed ads in our experiments in Section 5.

## 3. Ad Placement in the Presence of Interference

We describe ad placement in the presence of interference by a system of nonparametric structural equation models with independent errors (Pearl, 2009). The key characteristic of structural models is that they represent each variable as deterministic functions of their direct causes together with an unobserved exogenous noise term, which itself represents all causes outside of the model. Let *U* denote a variable capturing user intention which is unknown and hidden to the analyst. Given such intent, the user types a query, denoted by *C*, which is expressed as an unrestricted function of the intent *U* and a noise term ϵ_*c*_, denoted by *f*_*c*_(.). Upon observing the query, a set of ads are selected from the inventory, then online auction is run to determine winner ads to be displayed on the page. The *i*-th displayed ad is denoted by *X*_*i*_. The relation between *X*_*i*_ and *C* is captured by an unrestricted function *f*_*x*_*i*__(.) and the perturbation term ϵ_*xi*_. The block allocation of *i*-th ad is denoted by *A*_*i*_. The set of all impressed ads and the allocations are denoted by **X** and **A**, respectively (we suppress the indexing of pageviews for clarity). The information on *U*, **X**, **A**, along with the noise term ϵ_*yi*_, determines whether the *i*-th ad is clicked or not which is captured by *Y*_*i*_. The structural equation models are summarized as follows.


(1) $$\begin{array}{l} user\;intent\;U\;\leftarrow \;f_{u}(\epsilon_{u})\;\\ user\;query\;C\;\leftarrow \;f_{c}(U,\epsilon_{c})\;\\ i-th\;ad\;X_{i}\;\leftarrow \;f_{x_{i}}(C,\epsilon_{x_{i}})\\ i-th\;ad^{\prime }s\;allocation\;A_{i}\;\leftarrow \;f_{a_{i}}(X,\epsilon_{a_{i}})\\ i-th\;ad^{\prime }s\;click\;indication\;Y_{i}\;\leftarrow \;f_{y_{i}}(U,X,A,\epsilon_{y_{i}}) \end{array}$$

Note that in the above display, when allocating the *i*-th ad to Top or Bottom, we are not only considering the corresponding features of the ad itself, but also features of other ads on the page, hence the entire array of **X** is acting as causes of *A*_*i*_. Similarly, we allow for the entire vector of **A** and array of **X** to influence *Y*_*i*_. These equation capture the interference mechanism in ad placement. In the absence of interference, the above equations simplify by replacing the allocation structural equation with *A*_*i*_ ← *f*_*a*_*i*__(*X*_*i*_, ϵ_*a*_*i*__) and the click indication structural equation with *Y*_*i*_ ← *f*_*y*_*i*__(*U, X*_*i*_, *A*_*i*_, ϵ_*y*_*i*__).

**Figure 1:**
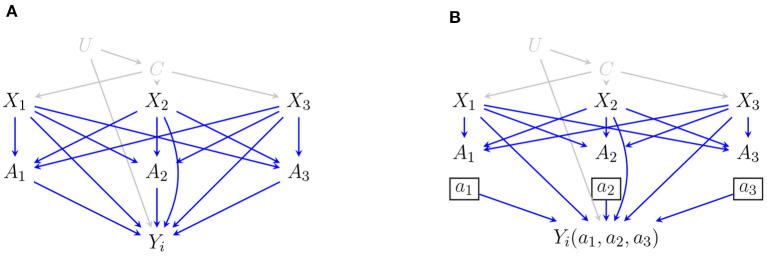
**(A)** DAG representation of the SEM in Equation (1) for a pageview with three impressed ads (the independent error terms are omitted from the graph for simplicity). **(B)** The corresponding SWIG where we intervene on *A* and set the block allocations (*A*_1_, *A*_2_, *A*_3_) to (*a*_1_, *a*_2_, *a*_3_).

Causal relationships are often represented by graphical causal models (Spirtes et al., 2000; Pearl, 2009). Such models generalize independence models on directed acyclic graphs (DAGs) to also encode conditional independencies on counterfactual variables (Richardson and Robins, 2013). A DAG G(V) consists of a set of nodes *V* connected through directed edges such that there are no directed cycles. We will abbreviate *G*(*V*) as simply *G*, when the vertex set is clear from the given context. Statistical models of a DAG *G* are sets of distributions that factorize as *p*(*V*) = ∏ _*V*_*i*_ ∈ *V*_ *p*[*V*_*i*_ ∣pa _*G*_(*V*_*i*_)], where pa_*G*_(*V*_*i*_) are the parents of *V*_*i*_ in *G*. The absence of edges between variables in *G*, relative to a complete DAG entails conditional independence facts in *p*(*V*). These can be directly read off from the DAG *G* by the well-known d-separation criterion (Pearl, 2009). That is, for disjoint sets *X, Y, Z*, the following *global Markov property* holds: (*X*_⊥⊥_d-sep_*Y* ∣ *Z*)*G*_ ⇒ (*X* ⊥⊥*Y* ∣ *Z*)_*p*(*V*)_. When the context is clear, we will simply use *X* ⊥⊥ *Y* ∣ *Z* to denote the conditional independence between *X* and *Y* given *Z*. The DAG representation of the structural (Equation 1) for a pageview with three impressed ads is shown in Figure 1A. For simplicity and to avoid cluttering the graph, we only depict the outcome of the *i*-th ad on the DAG and marginalize out all the other outcomes (since all the outcomes share the same set of parents). The statistical model of the DAG in Figure 1A, assuming all outcomes are included on the DAG, can be written as,


(2) $$\begin{array}{l} p(U,C,X,A,Y)\;=p(U)\times p(C|U)\times \prod {}_{i=1}^{3}\;\{p(X_{i}|C)\\ \;\times p(A_{i}|X)\times p(Y_{i}|U,X,A)\}. \end{array}$$

As we mentioned earlier, the user intent is unmeasured. We further restrict our attention to ad-specific features and leave the query-specific features aside. In other words *U* and *C* are both treated as latent. We highlight this in Figure 1A by coloring both vertices and the relevant edges in gray. In this case, the joint distribution over observed variables **X, A, Y** and latent variables *U, C* is said to be Markov relative to a hidden variable DAG. There may be infinitely many hidden variable DAGs that imply the same set of conditional independencies on the observed margin, i.e., *p*(**X, A, Y**). It is typical to use a single acyclic directed mixed graph that entails the same set of equality constraints as this infinite class; see Verma and Pearl (1990) and Richardson et al. (2017) for more details.

### 3.1. Sources of Interference in Ad Placement

In order to better understand the interference behavior among ads, we need to identify the causal mechanisms that give rise to such behaviors. Looking at our causal model in Figure 1A, we allow for two distinct pathways through which other ads influence *Y*_*i*_. One is direct pathways such as *X*_*j*_ → *Y*_*i*_ and *A*_*j*_ → *Y*_*i*_. This type of interference is called *direct interference*. As an example, suppose a low quality ad (determined by various scores) is placed in the Top. The poor quality of this ad may shape the user's opinion about the sorted search results in negative ways, preventing them from clicking on further ads. Similarly, placing a high quality ad in the Top may convince the user to return and explore more ads. Other pathways by which outcomes of different ads could be related are ones that go through the common unmeasured confounders and account for marginal dependencies between *Y*_*i*_ and *Y*_*j*_. An example of this marginal dependency is through user intent *U*, *Y*_*j*_ ← *U* → *Y*_*i*_. This type of interference is called *interference by homophily* (Shalizi and Thomas, 2011). Accounting for homophily makes our framework more practical as it allows for unmeasured confounders to influence multiple outcomes simultaneously. For a discussion on graphical representations of different sources of interference, see Ogburn and VanderWeele (2014).

The third type of interference that we account for is called *allocational interference*. In allocational interference, the interactions among units are modeled according to their corresponding group assignments. Through interactions within a group, units' characteristics may affect one another. This type of interference is well-suited for our purposes since each pageview is divided into non-overlapping blocks (Top and Bottom), and we can simply treat each block as a single group of ads. In our setting, treatment allocates each ad to a single block (randomly or given covariates *X*), and the outcome of the ad is affected by which other ads are allocated to the same block. We call this behavior *block-level interference*. We can also imagine a scenario where the outcome of an ad is affected by the ads that are **not** allocated to the same block. In other words, ads could potentially interact across blocks. We call this *cross-block interference*. As an example, moving a high quality ad to the Bottom may improve the perception of other ads in the Bottom and yield higher clicks on these ads. On the other hand, it may also affect the click yields of ads in the Top by drawing attention away from these ads, resulting in cross-block interactions. In order to formalize the block-level interference and cross-block interference, we split **X** into two disjoint sets: one that contains block-level information, denoted by **X**^*b*^, and one that contains information outside the block, denoted by **X**^*c*^. For the *i*-th positioned ad, we define two disjoint sets:


Xib={Xj∈X  s.t.  Aj=Ai}={I(Aj=Ai)×Xj, ∀j=1,…,m},Xic ={Xk∈X  s.t.  Aj=Ai}={I(Aj=Ai)×Xj, ∀j=1,…,m}.

We modify the structural equations for *Y_i_* in (Equation 1) to directly account for the allocational interference in our framework by simply replacing *f*_*y*_*i*__(*U*, **X, A**, ϵ_*y*_*i*__) with $f_{y_{i}}(U,X_{i}^{b},X_{i}^{c},\epsilon_{y_{i}}).$ Note that both ${\text{X}}_{i}^{b}$ and ${\text{X}}_{i}^{c}$ depend on the treatment rule *A* by construction. The function *f_yi_* can take a nonlinear or a linear form. For illustration, assume *f*_*y*_*i*__ is linear in parameters. Therefore, we have:


Yi ← ∑j=1mγj×I(Aj=Ai)×Xj+ηj×I(Aj=Ai)×Xj+ϵyi

In the above equation, γ_*j*_ controls the block-level influence of *X*_*j*_ on the *i*-th ad if *X*_*j*_ is in the same block as *X*_*i*_, otherwise the influence is controlled by the parameter η_*j*_. If η_*j*_ = 0, ∀*j*, then this implies that there is no cross-block interference and blocks are independent. If η_*j*_ = γ_*j*_, ∀*j*, then this implies that there is no allocational interference. In other words, interactions within blocks and across blocks are modeled exactly the same and therefore the notion of “groups” is ruled out.

## 4. Interference Effects Among Ads

Structural equation models, such as the one in display (1), enable us to determine the response of variables to interventions through incorporating knowledge of the functional dependencies between variables. For instance, intervening on the block allocation of the *i*-th ad would fix the value of *A*_*i*_ to *a*_*i*_, and would transform descendants of *A*_*i*_ to counterfactual variables of the form *V*(*a*_*i*_). Under an intervention that sets *A* to *a*, the structural (Equation 1) are modified as follows:


(3) $$\begin{array}{l} \;A_{i}\;\leftarrow \;a_{i},\;\forall i=1,\ldots ,m,\text{ and }\\ Y_{i}(a)\;\leftarrow \;f_{y_{i}}(U,X,a,\epsilon_{y_{i}}),\;\forall i=1,\ldots ,m. \end{array}$$

Interventions can be directly applied to the causal graph through a node-splitting operation where random variables in *A* are split into two parts: a random part that takes all the incoming edges and a fixed part that takes all the outgoing edges. The resulting graph is called a single-world intervention graph (SWIG) which encodes counterfactual independencies associated with the intervention (Richardson and Robins, 2013). Given the causal model in Figure 1A, we obtain the corresponding SWIG in Figure 1B after performing the intervention described in display (Equation 1).

### 4.1. Causal Effects of Interest

We set block allocation as our treatment of interest, and based on the prior literature, consider several causal effects that are of particular interest in ad placement systems.

1. *Unit-level effect*: defined as the effect of modifying an ad's block allocation on its clickability but holding the block allocations of other ads fixed. Assume we have a fixed allocation rule **a**, and we are interested in moving the *i*-th ad from block *a*′ to *a*″, i.e., altering the *i*-th element of *a* and allowing the other ads to follow the rule **a**_−*i*_. Then the unit-level effect is quantified via

$${\text{UE}}_{i}(a^{\prime },a^{\prime \prime },a)=\;𝔼[Y_{i}(a^{\prime },a_{-i})]-\;𝔼[Y_{i}(a^{\prime \prime },a_{-i})].$$

*2. Spillover effect*: defined as the effect of holding an ad's block allocation fixed but modifying the block allocations of other ads on the pageview. Assume we are interested in comparing two allocation rules **a′** and **a″** where the *i*-th element in each rule is fixed to *a*. Then the spill-over effect is quantified via

$${\text{SE}}_{i}(a,a^{\prime },a^{\prime \prime })=\;𝔼[Y_{i}(a,{a^{\prime }}_{-i})]-\;𝔼[Y_{i}(a,{a^{\prime \prime }}_{-i})].$$

*3. Overall effect*: defined as the effect of allocation rule *a* vs. *a*′ on the outcome of the *i*-th ad, which can be quantified via

$${\text{OE}}_{i}(a,a^{\prime })=\;𝔼[Y_{i}(a)]-\;𝔼[Y_{i}(a^{\prime })].$$

*4. Average overall effect*: defines as a pageview-level comparison of two different allocation rules. This would require an average over all the overall effects computed on a single pageview, i.e.,

$$\text{AOE}(a,a^{\prime })=\frac{1}{m}\;\sum_{i=1}^{m}\;𝔼[Y_{i}(a)]-\;𝔼[Y_{i}(a^{\prime })].$$

### 4.2. Identification Assumptions

Counterfactuals cannot in general be identified from data alone, and require assumptions. It is straightforward to see that all the effects described above involve counterfactual mean contrasts of the form 𝔼[*Y*_*i*_(**a**)]. Thus if we can identify this counterfactual mean, all the effects described are identifiable. In order to identify the counterfactual mean 𝔼[*Y*_*i*_(**a**)], we make the following three assumptions: (i) *Allocational consistency:* *Y*_*i*_(**a**) = *Y*_*i*_ if **A** = **a**, which means the potential outcome agrees with the observed outcome when the allocational intervention agrees with the observed allocations, (ii) *Positivity:* *p*(**A** = **a** ∣ **X** = **x**) > 0, ∀ **a** ∈𝔛 _**A**_ and ∀ **x** ∈𝔛 _**X**_, and (ii) *Network conditional ignorability:* *Y*_*i*_(**a**)⊥⊥**A**∣**X**, which means all the common confounders between each *A*_*j*_∈**A** and *Y*_*i*_ are measured.

Consistency and positivity assumptions are standard in causal inference (with or without the presence of interference). Even though, the no-unmeasured confounder assumption is also a common assumption in the literature, see Hudgens and Halloran (2008), Tchetgen and VanderWeele (2012), and Ogburn and VanderWeele (2014) for examples in the context of interference, this assumption is often untestable. In practice, we may either rely on domain knowledge to argue for the conditional ignorability assumption, or we can conduct a sensitivity analysis to know whether, and to what extent, the conclusions are robust to potential unmeasured confounding (Robins et al., 2000; Scharfstein et al., 2021). Fortunately, given the ad placement setup, described via the structural equations in display (1) and illustrated via the DAG in Figure 1A, we know the observed set *X* is fully responsible for deciding the allocations. Thus, the network conditional ignorability assumption still holds even in the presence of unmeasured confounders *U*, e.g., the use intent. Further, as mentioned previously, we can exclude the observed queries, collected in *C*, from the conditioning set as such factors do not play a direct role in neither choosing the allocations nor the final observed clicks. Using d-separation rules (Pearl, 2009), we can read off the independence between allocations *A* and counterfactual variable *Y*_*i*_(**a**) (conditioned on *X*) from the corresponding SWIG shown in Figure 1B.

Given the structural equation model described in Equation (1), the represented causal model in Figure 1A, and the corresponding SWIG in Figure 1B, we can easily verify that network conditional ignorability holds in our model. By rules of d-separation, all the paths from *Y*_*i*_(**a**) to each *A*_*j*_ is blocked by conditioning on **X**. Under the aforementioned assumptions, the identifying functional for 𝔼[*Y*_*i*_(**a**)] is then obtained as follows,


(4) $$𝔼[Y_{i}(a)]=\;𝔼[\;𝔼[Y_{i}|A=a,X]],$$

where the outer expectation is taken with respect to the marginal distribution over *X*, i.e., *p*(**X**). For a general theory describing when causal inference with interference is possible, interested readers can refer to Sherman and Shpitser (2018).

### 4.3. Estimation of Causal Effects

We set our target of inference to be ψ = 𝔼[*Y*_*i*_(**a**)] which is identified via (4). There are several ways of estimating this identified functional (e.g., G-computation methods, inverse probability weighting estimators, etc). In our experiments, we use the *augmented inverse probability weighting* (AIPW) estimator, given as


(5) $$\begin{array}{l} {\hat{\psi }}_{\text{aipw}}=\frac{1}{N}\sum_{n=1}^{N}[\frac{I(A_{n}=a)\times (Y_{in}-𝔼[Y_{in}|A=a,X_{n};{\hat{\alpha }}_{y}])}{\prod_{i=1}^{m}\;p(A_{in}=a_{i}|X_{n};{\hat{\alpha }}_{a})}\\ \;+\;𝔼[Y_{in}|A=a,X_{n};{\hat{\alpha }}_{y}]], \end{array}$$

where ${\hat{\alpha }}_{y}$ and ${\hat{\alpha }}_{a}$ are MLE estimates of the parameters in the outcome regression model 𝔼[*Y* ∣ **A**, **X**] and propensity models *p*(*A*_*i*_ ∣ **X**), respectively. The above estimator is consistent *if and only if* either the propensity scores or the outcome regression models are correctly specified. This property is known as *doubly robust*. For a more general discussion of semiparametric doubly robust estimators of average causal effects in presence of unmeasured confounders, see Bhattacharya et al. (2020a). An alternative approach is to use targeted maximum likelihood estimators (Van der Laan et al., 2007), that use an ensemble of machine learning models. We leave the exploration of TMLE to future work.

### 4.4. Verifying and Learning Causal Structure

Throughout the paper, we assumed a known causal structure for the ad placement system. To verify the correctness of our presumed causal structure, we adapt structure learning algorithms to learn the underlying mechanisms that give rise to interference. There is a rich literature on model selection from observational data in the context of causal inference with no interference (Spirtes et al., 2000). This includes constraint-based algorithms such as PC (Spirtes et al., 2000; Colombo and Maathuis, 2014), score-based algorithms such as GES (Chickering, 2002), and continuous optimization based algorithms such as the ones in and Bhattacharya et al. (2020b). Bhattacharya et al. (2019) provided a novel algorithm for model selection when units are related through a network of dependencies that can be modeled using a chain graph (Lauritzen, 1996). However, in our context, dependencies are best modeled using DAGs with hidden variables. There exist (conditional independence) constraint-based algorithms such as *fast causal inference* (FCI) and variations of it, such as GFCI and RFCI, that tackle the model selection problem in the presence of unmeasured confounders.

Click yields are the primary target of interest. Hence, we adapt the FCI algorithm in order to learn the “causal parents” of each *Y*_*i*_. We do this by performing a pre-processing step on the data, where each row corresponds to the information we collect on a single pageview, in order to account for block-level and cross-block interference. As an example, consider pageviews with three impressed ads where we are interested in finding the causal parents of the outcome in the first positioned ad, i.e., *Y*_1_. We pre-process the data as follows: For each row, we evaluate the variables in *X*_*j*_ to zero if *A*_*j*_ = *A*_1_, for *j* = 1, 2, 3. We call this pre-processed data *D*_1_. We then evaluate the variables in *X*_*j*_ to zero if *A*_*j*_ ≠ *A*_1_, for *j* = 2, 3. We call this pre-processed data *D*_2_. We then append *D*_2_ to *D*_1_, column-wise and pass this data to the FCI algorithm. Additional knowledge, such as causal ordering, can be incorporated in the procedure. The FCI algorithm then returns a partial ancestral graph (Zhang, 2008) as the Markov equivalence class. The partial ancestral graph corresponds to a set of ancestral acyclic directed mixed graphs (Richardson and Spirtes, 2002) that agree on conditional independence constraints on the observed data distribution. Under standard assumptions, that the true model can be represented via an ancestral graph and faithfulness, (asymptotically) FCI and hence our modification of it returns a Markov equivalence class that contains the true underlying model.

Here, we are working under a partial interference framework, where we model only interference within pageviews and exclude temporal dependence across pageviews. This means the search result pages are iid, but the ads inside each pageview do interact. Using the above description, we adapt the original FCI algorithm that assumes iid data to our framework for learning causal structures.

## 5. Experiments

In this section, we illustrate the utility of our formalization of the ad interference problem through four separate experiments using Bing PC traffic: (i) estimating the counterfactual mean under interference as described in Section 4, (ii) identifying causally relevant features through structure learning, (iii) comparing click prediction models with and without accounting for interference, and (iv) evaluating the performance of models with interfernece on layouts that do not appear in the training data. For training and validation purposes, we used data from the first 2 weeks of June in 2020. The test data comes from the first 2 weeks of July in the same year. We use random forest classifiers for fitting the propensity score and the outcome regression models.

We focused on two types of pageviews: *positive pageviews*, i.e., pageviews with at least one observed click (corresponding to users with an “ad frame of mind" who are more likely to click on an ad), and *balanced pageviews*, i.e., pageviews with positive and zero-clicked views. This scenario captures a more realistic view. We used AIPW to estimate the counterfactual mean 𝔼[*Y*_*i*_(**a**)] and ran our experiments on pageviews with 3, 4, and 5 number of impressed ads.

### 5.1. Calculation of Interference Effects

Recall that each allocation rule can be represented via a binary vector **a** = (*a*_1_, …, *a*_*m*_); e.g., when *m* = 3, the allocation (1, 1, 1) corresponds to a scenario where all three ads are shown in the Top block. As mentioned in the preliminaries, ads are indexed according to the order in which they appear on the page. This indexing scheme restricts the state space of all possible allocation rules. For instance, an allocation like (0, 1, 1) where the first positioned ad is placed at the Bottom and the rest are on Top is ill-defined and therefore excluded from the set of possible allocation rules.

**Figure 2:**
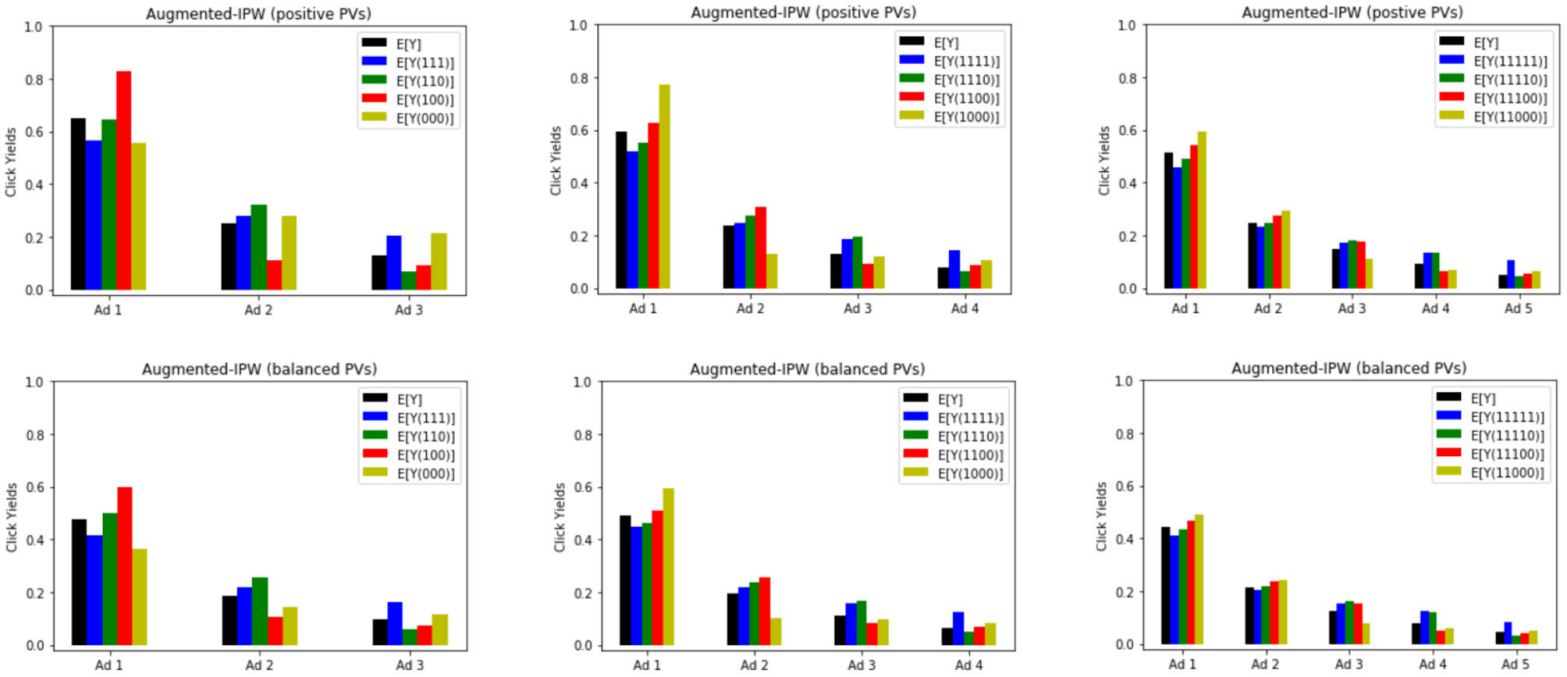
Estimates of 𝔼[*Y*_*i*_(a)] for all possible allocations using AIPW on pageviews with 3, 4, 5 impressed ads.

We use the AIPW estimator to compute the counterfactual mean 𝔼[*Y*_*i*_(**a**)] under all possible allocation rules for *a*. The results are shown in Figure 2. The layout that yields the highest click for each position on the pageview corresponds to the tallest bar on each plot. For instance for *m* = 3, the first positioned ad benefits the most from being the sole ad in the Top block, i.e., 𝔼[*Y*_1_(1, 0, 0)]>𝔼[*Y*_1_(**a**)], ∀**a**≠(1, 0, 0). However, the corresponding optimal layout for the first positioned ad is not coherent with the optimal layout of other ads. For instance, the second positioned ad benefits the most from being on the Top block as well. On the other hand, the last positioned ad benefits slightly more when all ads are placed at the Bottom. In order to find a coherent optimal layout yielding the highest number of overall clicks, we need to compare the average click response over all positions on the pageview, i.e., the average overall effect $\frac{1}{m}\overset{m}{\underset{i=1}{\sum }}\;𝔼\left[Y_{i}\left(\text{a}\right)\right],$ for all possible *a*.

Estimated values for all the counterfactual means (*m* = 3 with positive pageviews) are reported in Table 1 along with the corresponding 95% confidence intervals.Results on *m* = 4, 5 with the two types of pageviews are provided in Table 2. Additional information on frequencies of allocations are reported in Table 3. We can use these tables to compute various effects that were discussed in the previous section. For instance with *m* = 3, the following contrast gives us the unit-level effect for *Y*_2_ under allocation rule **a** = (1, 0, 0): UE_2_(1, 0, **a**) = 𝔼[*Y*_2_(1, 1, 0)−*Y*_2_(1, 0, 0)] = 0.32−0.11 = 0.21 (±0.004). This number quantifies the effect on clickability of the 2nd ad if we (hypothetically) moved it from Top to Bottom, while the 1st ad is kept on Top and the 3rd one is kept at Bottom. The spillover effect under allocation rules **a** = (1, 0, 0) and **a**′ = (1, 1, 1) is given by ${\text{SE}}_{2}(1,a\prime ,a)=𝔼[Y_{2}(1,1,1)-Y_{2}(1,1,0)]=0.28-0.32=-0.04\;(\pm 0.006).$ This number quantifies the effect on clickability of the 2nd ad if we changed the layout from *a*′ to *a*, while keeping the 2nd ad fixed on Top. The overall effect of *a*′ vs. **a**, i.e., ${\text{OE}}_{2}(a\prime ,a)=𝔼[Y_{2}(1,1,1)-Y_{2}(1,0,0)]$ is equal to the sum of UE and SE which is 0.17 (±0.007). Using Table 2, we can also compare the performance of each layout in terms of overall click yields. The results are provided in Table 4.

**Table 1:** Estimated values for the counterfactual mean 𝔼[*Y*_*i*_(a)] for all possible *a*, along with the 95% confidence intervals.

	**𝔼[*Y*_*i*_(1, 1, 1)]**	***𝔼*[*Y*_*i*_(1, 1, 0)]**	**𝔼[*Y*_*i*_(1, 0, 0)]**	**𝔼[*Y*_*i*_(0, 0, 0)]**	***𝔼*[*Y*_*i*_]**
1st ad	0.57 ± 0.006	0.64 ± 0.004	0.83 ± 0.004	0.56 ± 0.005	0.65
2nd ad	0.28 ± 0.007	0.32 ± 0.005	0.11 ± 0.003	0.28 ± 0.005	0.25
3rd ad	0.20 ± 0.006	0.07 ± 0.002	0.09 ± 0.003	0.21 ± 0.005	0.13

*The observed 𝔼[Y_i_] is reported on the last column (positive pageviews with m = 3)*.

**Table 2:** Estimation of counterfactual 𝔼[*Y*_*i*_(a)] along with 95% confidence interval.

	**Scenarios**		**Observed mean**	**0 at Bottom**	**1 at Bottom**	**2 at Bottom**	**3 at Bottom**
*m* = 3	Positives	*Y*_1_	0.65	0.57 ± 0.006	0.64 ± 0.004	0.83 ± 0.004	0.56 ± 0.005
		*Y*_2_	0.25	0.28 ± 0.007	0.32 ± 0.005	0.11 ± 0.003	0.28 ± 0.005
		*Y*_3_	0.13	0.20 ± 0.006	0.07 ± 0.002	0.09 ± 0.003	0.21 ± 0.005
	Balanced	*Y*_1_	0.48	0.41 ± 0.006	0.50 ± 0.004	0.60 ± 0.004	0.36 ± 0.008
		*Y*_2_	0.18	0.22 ± 0.008	0.25 ± 0.004	0.10 ± 0.003	0.15 ± 0.011
		*Y*_3_	0.10	0.16 ± 0.007	0.06 ± 0.002	0.07 ± 0.003	0.11 ± 0.011
*m* = 4	Positives	*Y*_1_	0.59	0.52 ± 0.010	0.55 ± 0.006	0.63 ± 0.006	0.77 ± 0.007
		*Y*_2_	0.24	0.25 ± 0.007	0.27 ± 0.004	0.31 ± 0.005	0.13 ± 0.004
		*Y*_3_	0.13	0.18 ± 0.006	0.20 ± 0.004	0.09 ±0.003	0.12 ± 0.004
		*Y*_4_	0.08	0.14 ± 0.005	0.06 ± 0.002	0.09 ± 0.002	0.11 ± 0.003
	Balanced	*Y*_1_	0.49	0.45 ± 0.007	0.46 ± 0.005	0.51 ± 0.005	0.59 ± 0.009
		*Y*_2_	0.20	0.22 ± 0.004	0.24 ± 0.003	0.26 ± 0.004	0.10 ± 0.003
		*Y*_3_	0.11	0.16 ± 0.004	0.17 ± 0.003	0.08 ± 0.002	0.09 ± 0.003
		*Y*_4_	0.06	0.13 ± 0.003	0.05 ± 0.001	0.07 ± 0.002	0.08 ± 0.003
*m* = 5	Positives	*Y*_1_	0.51	0.46 ± 0.009	0.49 ± 0.006	0.54 ± 0.006	0.59 ± 0.018
		*Y*_2_	0.24	0.23 ± 0.005	0.25 ± 0.004	0.27 ± 0.004	0.30 ± 0.010
		*Y*_3_	0.15	0.17 ± 0.005	0.18 ± 0.004	0.18 ± 0.004	0.11 ± 0.006
		*Y*_4_	0.09	0.13 ± 0.004	0.13 ± 0.003	0.06 ± 0.002	0.07 ± 0.005
		*Y*_5_	0.05	0.11 ± 0.004	0.04 ± 0.002	0.05 ± 0.002	0.06 ± 0.005
	Balanced	*Y*_1_	0.44	0.41 ± 0.011	0.43 ± 0.006	0.47 ± 0.006	0.49 ± 0.019
		*Y*_2_	0.21	0.21 ± 0.006	0.22 ± 0.004	0.24 ± 0.004	0.24 ± 0.009
		*Y*_3_	0.13	0.15 ± 0.005	0.16 ± 0.003	0.15 ± 0.003	0.08 ± 0.005
		*Y*_4_	0.08	0.12 ± 0.005	0.12 ± 0.003	0.05 ± 0.002	0.06 ± 0.005
		*Y*_5_	0.04	0.08 ± 0.007	0.03 ± 0.002	0.04 ± 0.003	0.05 ± 0.009

*Each allocation a is represented by the number of ads that it places at the Bottom. For instance “1 at Bottom” corresponds to the allocation (1, 1, 0) when m = 3, (1, 1, 1, 0) when m = 4, and (1, 1, 1, 1, 0) when m = 5. Allocations with “4 at Bottom” and “5 at Bottom” do not appear in the time span we considered. The observed 𝔼[Y_i_] is reported on the second column*.

**Table 3:** Observed frequencies of allocations.

**Impressed ads and scenarios**		**0 at Bottom(%)**	**1 at Bottom(%)**	**2 at Bottom(%)**	**3 at Bottom(%)**
*m* = 3	Positives	36.9	31.7	22.6	8.8
	Balanced	32.5	29.4	22.9	15.1
*m* = 4	Positives	32.0	26.5	24.9	16.6
	Balanced	30.3	25.9	25.4	18.4
*m* = 5	Positives	29.3	27.2	23.5	20.0
	Balanced	28.5	26.7	23.8	21.1

*Allocations with “4 at Bottom” and “5 at Bottom” do not appear in the time span we considered*.

**Table 4:** Layout comparisons by reporting average overall counterfactual mean, i.e., $\frac{1}{m}\overset{m}{\underset{i=1}{\sum }}\;𝔼\left[Y_{i}\left(a\right)\right],$ for all possible allocations.

**Impressed ads and scenarios**		**Observed**	**0 at Bottom**	**1 at Bottom**	**2 at Bottom**	**3 at Bottom**
*m* = 3	Positives	0.3437	0.3495 ±1.2e-7	0.3460 ±1.4e-7	0.3442 ±1.58e-7	0.3499 ±1.91e-7
	Balanced	0.2526	0.2649 ± 2.0e-7	0.2713 ±2.3e-7	0.2577 ±2.7e-7	0.2079 ±5.0e-7
*m* = 4	Positives	0.2591	0.2727 ± 1.5e-7	0.2709 ±1.5e-7	0.2786 ±1.9e-7	0.2816 ±2.2e-7
	Balanced	0.2140	0.2374 ±1.3e-7	0.2294 ±1.8e-7	0.2275 ±2.2e-7	0.2171 ±3.1e-7
*m* = 5	Positives	0.2087	0.2211 ±1.3e-7	0.2186 ±1.6e-7	0.2227 ±1.8e-7	0.2260 ±3.2e-7
	Balanced	0.1807	0.1945 ±2.3e-7	0.1926 ±2.6e-7	0.1897 ±3.0e-7	0.1836 ±5.1e-7

### 5.2. Learning the Causal Structure Using FCI

In this part of the experiment, we use data to learn the parents of each outcome for all ads on the pageview; while allowing for both block-level and cross-block interference. We preprocess the data as described in Section 4.4, and use the implementation of the FCI algorithm in the Tetrad software^1^. Independence tests are performed using kernel conditional independence tests (Zhang et al., 2012) with a significance level of 0.01. On each pageview, we collect *m* × 22 different features. Neither plotting the learned graph nor enlisting all parental sets is relevant to the point we like to deliver here. Our primary objective is to show that for a particular positioned ad, features from other ads on the pageview (not necessarily from the same block even) are directly relevant to the clickability of the ad. In order for us to report the results in a more concise and clear way, we divide the ad-specific features into four distinct categories: (a) Calculated scores, such as *PClick, PDefect, Relevance score, etc*., (b) Decorative features, such as *Twitter information, links, and ratings*, (c) Geometric features, such as *line counts, pixel heights, pixel heights from top of the block*, and (d) match type information. We found out that the parent set of each *Y*_*i*_ contains at least one variable in each category of features from a different ad; providing further evidence for the presence of interference among ads. In our extended set of experiments, we learned that Decorative features are more influential on pageviews with higher number of impressed ads. Please refer to the appendix for more experiments.

We further designate a fifth category (e) for collection of exogenous features that are layout-specific, such as *ad counts*. For each scenario, we report what categories the causally relevant features belong to in Table 5. For each positioned ad, the influence of other ads on the pageview are spread over multiple categories of features. Calculated scores and geometric features are influential in clickability across all scenarios and pageviews with different number of impressed ads.

**Table 5:** Using FCI procedure to learn the structure of our model, this table reports what categories the causally relevant features belong to.

	**Scenarios**		**Calculated scores**	**Decorative features**	**Geometric features**	**Match type**	**Exogenous features**
*m* = 3	Positives	*Y*_1_	✓		✓	✓	
		*Y*_2_	✓		✓	✓	
		*Y*_3_	✓		✓	✓	
	Balanced	*Y*_1_	✓		✓		✓
		*Y*_2_	✓		✓		✓
		*Y*_3_	✓		✓	✓	✓
*m* = 4	Positives	*Y*_1_	✓		✓		✓
		*Y*_2_	✓		✓	✓	
		*Y*_3_	✓		✓	✓	
		*Y*_4_	✓	✓	✓	✓	
	Balanced	*Y*_1_	✓		✓		
		*Y*_2_	✓		✓	✓	
		*Y*_3_	✓		✓	✓	
		*Y*_4_	✓	✓	✓	✓	
*m* = 5	Positives	*Y*_1_	✓	✓	✓	✓	✓
		*Y*_2_	✓	✓	✓	✓	
		*Y*_3_	✓		✓		
		*Y*_4_	✓	✓	✓	✓	
		*Y*_5_	✓		✓	✓	
	Balanced	*Y*_1_	✓	✓	✓	✓	
		*Y*_2_	✓	✓	✓	✓	
		*Y*_3_	✓		✓		
		*Y*_4_	✓	✓	✓		
		*Y*_5_	✓		✓	✓	

### 5.3. Improvements in Click Prediction

Given the set of experiments described above, we have more evidence to believe that interference *does* exist among ads. This was shown through both finding effects that are away from zero and learning causally relevant features that originate from other ads on the pageview. We now leverage this knowledge to better estimate the click yields. We considered fitting 5 different sets of models:

*(1) (Baseline)* model where samples are assumed to be independent, i.e., fitting *p*(*Y*_*i*_ = 1∣*L, X*_*i*_),
*(2) (Block-level interference)* model where we allow for block-level interactions like *I*(*A*_*j*_ = *A*_*i*_) × *X*_*j*_, i.e., *p*[*Y*_*i*_ = 1∣*L, X*_*i*_, *I*(*A*_*j*_ = *A*_*i*_) × *X*_*j*_],
*(3) (Block-level and cross-block interference)* model where in addition to block-level interactions we allow for cross-block interactions, i.e., *p*[*Y*_*i*_ = 1∣*L, X*_*i*_, *I*(*A*_*j*_ = *A*_*i*_) × *X*_*j*_, *I*(*A*_*k*_≠*A*_*i*_) × *X*_*k*_],
*(4) (Full graph)* with no block decomposition, i.e., *p*(*Y*_*i*_ = 1∣*L, X*), and
*(5) (FCI parents)* model where we use the parents of *Y*_*i*_ in the graph that FCI outputs, i.e., fitting *p*[*Y*_*i*_ = 1∣pa(*Y*_*i*_)].

**Figure 3:**
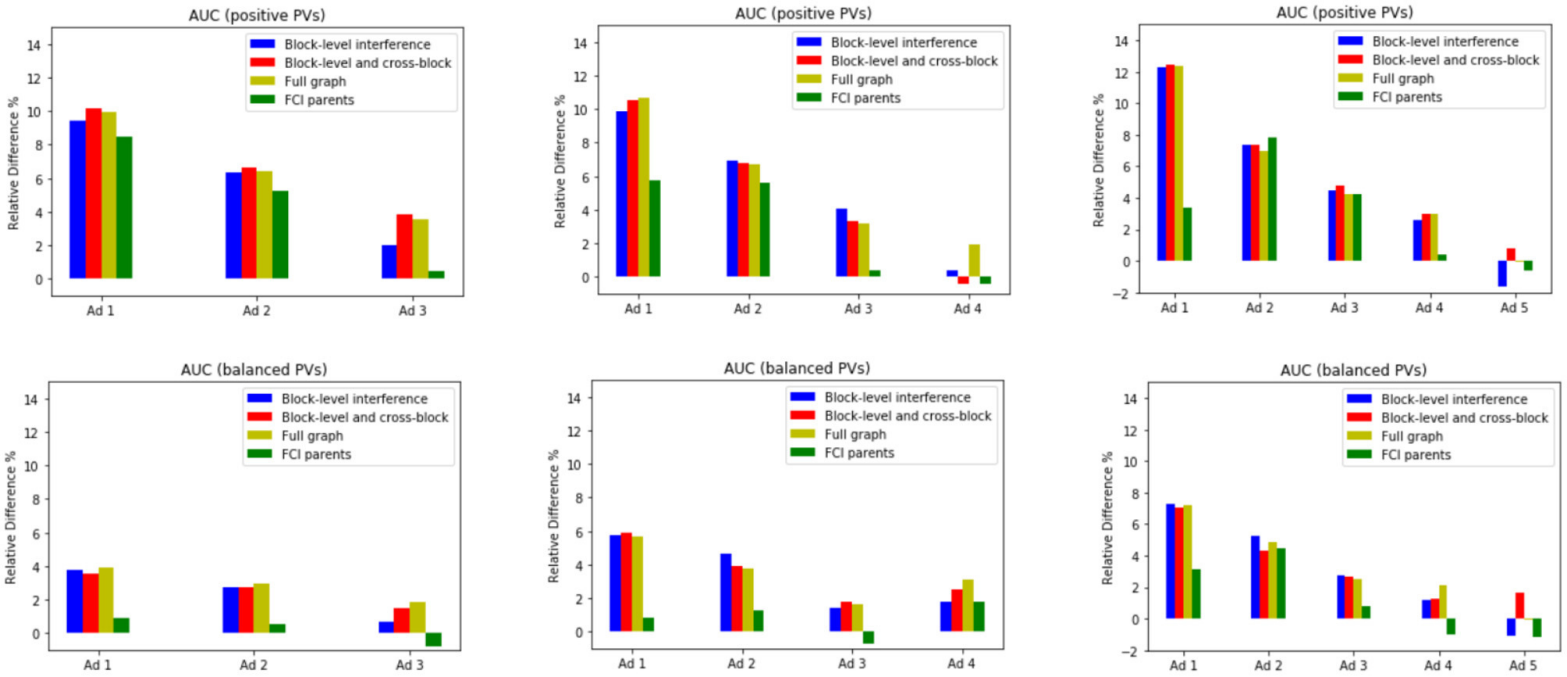
Relative difference (in percentage) in AUCs with respect to the baseline model.

We report relative improvements in area under the curve over the baseline in Figure 3. All methods that account for interference show improvement over the baseline, demonstrating the utility of our formalization. It is also worth noting that the performance gains are greater for higher positioned ads compared to lower ones.

### 5.4. Performance in Unseen Layouts

**Figure 4:**
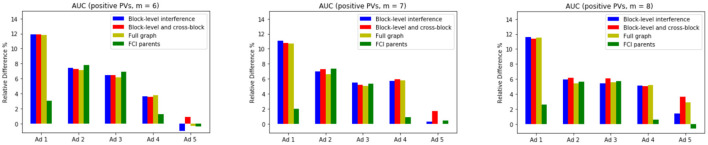
Relative difference (in percentage) in AUCs with respect to the baseline model in unseen layouts.

We evaluate the performance of our models with interference on layouts that do not appear in the training data. We limit our training data to pageviews with 5 impressed ads and test the models on pageviews that have more than 5 impressed ads. Figure 4 highlights the improvement of the proposed models on pageviews with 6, 7, and 8 impressed ads.

## 6. Conclusion

Despite the intuition that ads should not be scrutinized independently of one another, to the best of our knowledge, there has not been a formal analysis of interference in advertisement placement and sponsored search marketing. In this paper, we formalized the interference problem among ads using the language of causal inference and counterfactual reasoning. We proposed a framework to quantify the interference effects by posing a graphical causal model that accounts for potential underlying interference mechanisms. We described several causal effects that might be of interest in ad placement systems and discussed identification assumptions and estimation strategies for computing these effects. We further adapted the FCI procedure to learn the underlying mechanisms that give rise to interference and verify the correctness of our presumed causal structures.

In the partial interference framework, it is often assumed that the iid units are of the same size. The equivalent assumption we made is that pageviews have a fixed number of impressed ads. If sample size is not of concern, we can analyze each pageview of size *m* in isolation. However, in scenarios where data is scarce, we need alternatives to relax this restrictive assumption. One approach is through feature engineering where we first assume that only *k* nearest neighbors are interacting with the ad itself, a *Markov order of* *k* assumption if you will. We further need to assume the neighboring ads influence one another in the exact similar ways, a *parameter sharing* assumptions, if you will. Investigating such alternatives and exploring other approaches opens up an interesting direction for future work.

In this paper, we focused on the impressed ads on the search result page, and marginalized out the ads involved in the search engine auction. Incorporating the knowledge on how exactly the auction optimizer works on the entire set of candidate ads is important in determining the optimal layouts in presence of interference. We further restricted our attention to auctions that only yield two blocks on the final pageview. This can be simply relaxed by allowing for the allocation treatment to have a discrete state space. We can further group the ads that were not impressed and treat them as a separate block, and investigate their impact on the click yields of the other ads on the page.

## Data Availability Statement

The aggregated data supporting the conclusions of this article will be made available upon request. Further requests will be assessed on a case-by-case basis to ensure compliance with privacy agreements and other requirements. Requests to access the datasets should be directed to the corresponding author.

## Author Contributions

RN, DC, and EK contributed to conception and design of the framework. JP organized the database. RN performed the statistical analysis and wrote the first draft of the manuscript. All authors contributed to manuscript revision, read, and approved the submitted version.

## Conflict of Interest

JP, DC, and EK were employed by Microsoft Corporation. The research was conducted while RN was an intern at Microsoft Research.

## Publisher's Note

All claims expressed in this article are solely those of the authors and do not necessarily represent those of their affiliated organizations, or those of the publisher, the editors and the reviewers. Any product that may be evaluated in this article, or claim that may be made by its manufacturer, is not guaranteed or endorsed by the publisher.

## Footnotes

^1^ https://www.phil.cmu.edu/tetrad/publications.html

## References

[B1] Bajari P. Burdick B. Imbens G. W. Masoero L. McQueen J. Richardson T.. (2021). Multiple randomization designs. arXiv preprint arXiv:2112.13495. 10.48550/arXiv.2112.13495

[B2] Bayir M. A. Xu M. Zhu Y. Shi Y. (2019). “Genie: an open box counterfactual policy estimator for optimizing sponsored search marketplace,” in Proceedings of the Twelfth ACM International Conference on Web Search and Data Mining, 465–473.

[B3] Bhattacharya R. Nabi R. Shpitser I. (2020a). Semiparametric inference for causal effects in graphical models with hidden variables. arXiv preprint arXiv:2003.12659. 10.48550/arXiv.2003.12659

[B4] Bhattacharya R. Nagarajan T. Malinsky D. Shpitser I. (2020b). Differentiable causal discovery under unmeasured confounding. arXiv preprint arXiv:2010.06978. 10.48550/arXiv.2010.06978

[B5] Bhattacharya R. Malinsky D. Shpitser I. (2019). “Causal inference under interference and network uncertainty,” in Uncertainty in Artificial Intelligence: Proceedings of the... Conference. Conference on Uncertainty in Artificial Intelligence, volume 2019 (NIH Public Access). PMC6935347 31885520

[B6] Bisht K. Susan S. (2021). “Weighted ensemble of neural and probabilistic graphical models for click prediction,” in 2021 the 5th International Conference on Information System and Data Mining, 145–150.

[B7] Bottou L. Peters J. Quiñonero-Candela J. Charles D. X. Chickering D. M. Portugaly E.. (2013). Counterfactual reasoning and learning systems: the example of computational advertising. J. Mach. Learn. Res. 14, 3207–3260.

[B8] Cheng H. Cantú-Paz E. (2010). “Personalized click prediction in sponsored search,” in Proceedings of the Third ACM International Conference on Web Search and data Mining, 351–360.

[B9] Cheng H. Zwol R. V. Azimi J. Manavoglu E. Zhang R. Zhou Y.. (2012). “Multimedia features for click prediction of new ads in display advertising,” in Proceedings of the 18th ACM SIGKDD International Conference on Knowledge Discovery and Data Mining, 777–785.

[B10] Chickering D. M.. (2002). Optimal structure identification with greedy search. J. Mach. Learn. Res. 3, 507–554.

[B11] Colombo D. Maathuis M. H. (2014). Order-independent constraint-based causal structure learning. J. Mach. Learn. Res. 15, 3741–3782.35327862

[B12] Effendi M. J. Ali S. A. (2017). Click through rate prediction for contextual advertisment using linear regression. arXiv preprint arXiv:1701.08744. 10.48550/arXiv.1701.08744

[B13] Harshaw C. Sävje F. Eisenstat D. Mirrokni V. Pouget-Abadie J. (2021). Design and analysis of bipartite experiments under a linear exposure-response model. arXiv preprint arXiv:2103.06392. 10.48550/arXiv.2103.06392

[B14] Hill D. N. Moakler R. Hubbard A. E. Tsemekhman V. Provost F. Tsemekhman K. (2015). “Measuring causal impact of online actions via natural experiments: application to display advertising,” in Proceedings of the 21th ACM SIGKDD International Conference on Knowledge Discovery and Data Mining, 1839–1847.

[B15] Huang Y. Valtorta M. (2006). “Pearl's calculus of intervention is complete,” in Proceedings of the 22nd Conference on Uncertainty in Artificial Intelligence, 13–16.

[B16] Hudgens M. G. Halloran M. E. (2008). Toward causal inference with interference. J. Am. Stat. Assoc. 103, 832–842. 10.1198/016214508000000292 19081744 PMC2600548

[B17] Johari R. Li H. Liskovich I. Weintraub G. Y. (2022). Experimental design in two-sided platforms: an analysis of bias. Manag. Sci. 10.1287/mnsc.2021.4247

[B18] Lauritzen S. L.. (1996). Graphical Models. Oxford, UK: Clarendon.

[B19] Nabi R. Kanki P. Shpitser I. (2018). “Estimation of personalized effects associated with causal pathways,” in Uncertainty in Artificial Intelligence: Proceedings of the... Conference. Conference on Uncertainty in Artificial Intelligence, Vol. 2018 (NIH Public Access). PMC6330047 30643490

[B20] Nabi R. Malinsky D. Shpitser I. (2019). “Learning optimal fair policies,” in International Conference on Machine Learning (PMLR), 4674–4682. PMC6935348 31886463

[B21] Nabi-Abdolyousefi R.. (2015). Conversion rate prediction in search engine marketing (Ph.D. thesis).

[B22] Ogburn E. L. VanderWeele T. J. (2014). Causal diagrams for interference. Stat. Sci. 29, 559–578. 10.1214/14-STS501

[B23] Pearl J.. (2009). Causality. Cambridge: Cambridge University Press.

[B24] Pouget-Abadie J. Aydin K. Schudy W. Brodersen K. Mirrokni V. (2019). “Variance reduction in bipartite experiments through correlation clustering,” in 33rd Conference on Neural Information Processing Systems (NeurIPS 2019) (Vancouver, BC).

[B25] Pouget-Abadie J. Parkes D. C. Mirrokni V. Airoldi E. M. (2018). Optimizing cluster-based randomized experiments under a monotonicity assumption. arXiv preprint arXiv:1803.02876. 10.1145/3219819.3220067

[B26] Richardson T. S. Evans R. J. Robins J. M. Shpitser I. (2017). Nested Markov properties for acyclic directed mixed graphs. arXiv preprint arXiv:1701.06686. 10.48550/arXiv.1701.0668630983907

[B27] Richardson T. S. Robins J. M. (2013). “Single world intervention graphs (SWIGs): a unification of the counterfactual and graphical approaches to causality,” in Center for the Statistics and the Social Sciences, University of Washington Series. Working Paper (Washington, DC).

[B28] Richardson T. S. Spirtes P. L. (2002). Ancestral graph Markov models. Ann. Stat. 30, 962–1030. 10.1214/aos/1031689015

[B29] Robins J. M.. (1986). A new approach to causal inference in mortality studies with a sustained exposure period-application to control of the healthy worker survivor effect. Math. Model. 7, 1393–1512. 10.1016/0270-0255(86)90088-6

[B30] Robins J. M. Rotnitzky A. Scharfstein D. O. (2000). “Sensitivity analysis for selection bias and unmeasured confounding in missing data and causal inference models,” in Statistical Models in Epidemiology, the Environment, and Clinical Trials (Springer), 1–94.

[B31] Rubin D. B.. (1980). Randomization analysis of experimental data: the fisher randomization test comment. J. Am. Stat. Assoc. 75, 591–593. 10.2307/2287653

[B32] Scharfstein D. O. Nabi R. Kennedy E. H. Huang M.-Y. Bonvini M. Smid M. (2021). Semiparametric sensitivity analysis: unmeasured confounding in observational studies. arXiv preprint arXiv:2104.08300. 10.48550/arXiv.2104.08300 39400259

[B33] Shalizi C. R. Thomas A. C. (2011). Homophily and contagion are generically confounded in observational social network studies. Sociol. Methods Res. 40, 211–239. 10.1177/0049124111404820 22523436 PMC3328971

[B34] Shaparenko B. Çetin Ö. Iyer R. (2009). “Data-driven text features for sponsored search click prediction,” in Proceedings of the Third International Workshop on Data Mining and Audience Intelligence for Advertising, 46–54.

[B35] Sherman E. Shpitser I. (2018). “Identification and estimation of causal effects from dependent data,” in Advances in Neural Information Processing Systems, 9424–9435. 30643365 PMC6330046

[B36] Shpitser I.. (2013). Counterfactual graphical models for longitudinal mediation analysis with unobserved confounding. Cogn. Sci. 37, 1011–1035. 10.1111/cogs.12058 23899340

[B37] Shpitser I. Pearl J. (2012). Identification of conditional interventional distributions. arXiv preprint arXiv:1206.6876. 10.48550/arXiv.1206.6876

[B38] Shpitser I. Pearl J. (2006). “Identification of joint interventional distributions in recursive semi-Markovian causal models,” in Proceedings of the 21st National Conference on Artificial Intelligence.

[B39] Sobel M. E.. (2006). What do randomized studies of housing mobility demonstrate? causal inference in the face of interference. J. Am. Stat. Assoc. 101, 1398–1407. 10.1198/016214506000000636

[B40] Spirtes P. L. Glymour C. N. Scheines R. Heckerman D. Meek C. Cooper G.. (2000). Causation, Prediction, and Search. MIT Press.

[B41] Tchetgen E. J. T. VanderWeele T. J. (2012). On causal inference in the presence of interference. Stat. Methods Med. Res. 21, 55–75. 10.1177/0962280210386779 21068053 PMC4216807

[B42] Van der Laan M. J. Polley E. C. Hubbard A. E. (2007). Super learner. Stat. Appl. Genet. Mol. Biol. 6, 25. 10.2202/1544-6115.1309 17910531

[B43] Verma T. Pearl J. (1990). “Equivalence and synthesis of causal models,” in Proceedings of the 6th Conference on Uncertainty in Artificial Intelligence.

[B44] Wang X.. (2020). “A survey of online advertising click-through rate prediction models,” in 2020 IEEE International Conference on Information Technology, Big Data and Artificial Intelligence (ICIBA), Vol. 1, (Chongqing: IEEE), 516–521.

[B45] Xiong C. Wang T. Ding W. Shen Y. Liu T.-Y. (2012). “Relational click prediction for sponsored search,” in Proceedings of the Fifth ACM International Conference on Web Search and Data Mining, 493–502.

[B46] Yin D. Cao B. Sun J.-T. Davison B. D. (2014). “Estimating ad group performance in sponsored search,” in Proceedings of the 7th ACM International Conference on Web Search and Data Mining, 143–152.

[B47] Zeng S. Bayir M. A. Pfeiffer I. I. I. J. J Charles D. Kiciman E. (2021). “Causal transfer random forest: combining logged data and randomized experiments for robust prediction,” in Proceedings of the 14th ACM International Conference on Web Search and Data Mining, 211–219.

[B48] Zhang J.. (2008). On the completeness of orientation rules for causal discovery in the presence of latent confounders and selection bias. Artif. Intell. 172, 1873–1896. 10.1016/j.artint.2008.08.001

[B49] Zhang K. Peters J. Janzing D. Schölkopf B. (2012). Kernel-based conditional independence test and application in causal discovery. arXiv preprint arXiv:1202.3775. 10.48550/arXiv.1202.3775

[B50] Zhang Y. Dai H. Xu C. Feng J. Wang T. Bian J.. (2014). Sequential click prediction for sponsored search with recurrent neural networks. arXiv preprint arXiv:1404.5772. 10.48550/arXiv.1404.5772

